# Association of HIV status with sexual function in women aged 45–60 in England: results from two national surveys

**DOI:** 10.1080/09540121.2019.1653436

**Published:** 2019-08-14

**Authors:** Nasreen Toorabally, Catherine H. Mercer, Kirstin R. Mitchell, Mwenza Blell, Fiona Burns, Richard Gilson, Janine McGregor-Read, Sris Allan, Annemiek De Ruiter, Rageshri Dhairyawan, Julie Fox, Yvonne Gilleece, Rachael Jones, Nicola Mackie, Sharmin Obeyesekera, Frank Post, Iain Reeves, Melanie Rosenvinge, Jonathan Ross, Liat Sarner, Ann Sullivan, Anjum Tariq, Andrew Ustianowski, Caroline A. Sabin, Shema Tariq

**Affiliations:** a Institute for Global Health, University College London, London, UK; b NIHR Health Protection Research Unit in Blood Borne and Sexually Transmitted Infections, University College London, London, UK; c MRC/CSO Social and Public Health Sciences Unit, Institute of Health and Wellbeing, University of Glasgow, Glasgow, UK; d The Policy Ethics and Life Sciences (PEALS) Research Centre, School of Geography, Politics, and Sociology, Newcastle University, Newcastle, UK; e Ian Charleson Day Centre, Royal Free London NHS Foundation Trust, London, UK; f Positively UK, London, UK; g City of Coventry Health Centre (Integrated Sexual Health Services), Coventry and Warwickshire Partnership NHS Trust, Coventry, UK; h Harrison Wing, Guy’s & St Thomas’ NHS Foundation Trust, London, UK; i ViiV Healthcare, London, UK; j Outpatients East, Barking, Havering and Redbridge NHS Trust, Barking, UK; k Lawson Unit, Brighton & Sussex University Hospitals NHS Trust, Brighton, UK; l Kobler Outpatient Clinic, Chelsea and Westminster Hospital NHS Foundation Trust, London, UK; m The Wharfside Clinic, Imperial College Healthcare NHS Trust, London, UK; n Caldecot Centre, King's College Hospital NHS Foundation Trust, London, UK; o Jonathan Mann Clinic, Homerton University Hospital Foundation Trust, London, UK; p Trafalgar Clinic, Lewisham and Greenwich NHS Trust, London, UK; q Queen Elizabeth Hospital Birmingham HIV Clinic, University Hospital Birmingham NHS Foundation Trust, Birmingham, UK; r Grahame Hayton Unit, Barts NHS Trust, London, UK; s Wolverhampton Sexual Health Service, The Royal Wolverhampton NHS Trust, Wolverhampton, UK; t Department of Infectious Diseases, North Manchester General Hospital, Penine Acute Hospitals NHS Trust, Manchester, UK

**Keywords:** HIV, women, sexual function, menopause

## Abstract

Increasing numbers of women living with HIV are reaching their midlife. We explore the association of HIV status with sexual function (SF) in women aged 45–60 using two national cross-sectional surveys: the third British National Survey of Sexual Attitudes and Lifestyles (“Natsal-3”) and “PRIME”, a survey of women living with HIV attending HIV clinics across England. Both studies asked the same questions about SF that take account not only sexual difficulties but also the relationship context and overall level of satisfaction, which collectively allowed an overall SF score to be derived. We undertook analyses of sexually-active women aged 45–60 from Natsal-3 (*N *= 1228, presumed HIV-negative given the low estimated prevalence of HIV in Britain) and PRIME (*N *= 386 women living with HIV). Women living with HIV were compared to Natsal-3 participants using multivariable logistic regression (adjusting for key confounders identified *a priori*: ethnicity, ongoing relationship status, depression and number of chronic conditions) and propensity scoring. Relative to Natsal-3 participants, women living with HIV were more likely to: have low overall SF (adjusted odds ratio (AOR) 3.75 [2.15–6.56]), report ≥1 sexual problem(s) lasting ≥3 months (AOR 2.44 [1.49–4.00]), and report almost all 8 sexual problems asked about (AORs all ≥2.30). The association between HIV status and low SF remained statistically significant when using propensity scoring (AOR 2.43 [1.68–3.51]). Among women living with HIV (only), low SF was more common in those who were postmenopausal vs. Premenopausal (55.6% vs. 40.4%). This study suggests a negative association between HIV status and sexual function in women aged 45–60. We recommend routine assessment of SF in women living with HIV.

## Introduction

In the UK, approximately 10,500 women of potentially menopausal age (45–56 years) attended for HIV care in 2016, a five-fold increase over ten years (Z Yin, Public Health England, personal communication, 3 October 2017). Consequently, understanding the effects of the menopause on women living with HIV is important to inform HIV-related care.

Studies from the North America, Australia and Europe have shown that the menopause can contribute to poor sexual function in women without HIV (Avis et al., 2017; Dennerstein, Alexander, & Kotz, 2003; Mitchell et al., 2013), and is associated with declines across several domains including desire, lubrication and orgasm (Çayan et al., 2004; Dennerstein et al., 2003). Data from Britain’s third National Survey of Sexual Attitudes and Lifestyles (Natsal-3) reveal that almost one in ten female respondents aged 55–64 reported pain during sex, with this being associated with postmenopausal status (Mitchell et al., 2017).

Two European studies (both conducted over ten years ago) have found that between 25–50% of women living with HIV report sexual problems including poor lubrication, pelvic pain, and low sexual satisfaction (Bell, Richardson, Wall, & Goldmeier, 2006; Florence et al., 2004). An analysis of data from the US Women’s Interagency HIV Study found that women living with HIV were more likely to report sexual impairment compared to women without HIV, across all age groups (Wilson et al., 2010). Qualitative data reveal the importance of sexual pleasure for older women living with HIV, with diminished libido and vaginal dryness highlighted as particularly important (Taylor et al., 2017). Data on sexual function in older women living with HIV remain limited, and mainly originate from the US or Brazil. Postmenopausal declines in sexual function and activity have been shown among women living with HIV in some of these studies (as also seen in women without HIV) (Taylor et al., 2015; Wilson et al., 2010), but to the best of our knowledge no studies have looked at whether the impact of the menopause on sexual function differs by HIV status. Genitourinary symptoms are common, both peri and postmenopausally (Fantry, Zhan, Taylor, Sill, & Flaws, 2005; Ferreira et al., 2007; Lui-Filho et al., 2013); one study has found an association with HIV status (Miller et al., 2005). The only study to date to examine dyspareunia in menopausal women with and without HIV did not show any significant difference between the two groups (Valadares et al., 2014).

Using two large, national surveys, we aim to describe for the first time in England (i) the association between HIV status and sexual function in women aged 45–60, and (ii) whether any association between menopausal status and sexual function differs by HIV status.

## Methods

We undertook comparative analyses of data from sexually-active women aged 45–60 in England who participated in either Natsal-3, to provide a sample of sexually-active presumed HIV-negative women, or the PRIME (Positive TRansItions through the MEnopause) Study, a convenience survey undertaken in HIV clinics across England, providing a sample of sexually-active women living with HIV.

### Data sources

#### Natsal-3

Natsal-3 is a one of the largest studies of sexual behaviour and lifestyles internationally. It used a multi-stage, clustered and stratified probability sample design to invite men and women aged 16–74 years, living in private households in Britain, to participate (Erens et al., 2014). Data were collected between September 2010 and August 2012, and involved a combination of a face-to-face interview using computer-assisted personal interviewing and a self-completion format using computer-assisted self-interviewing (Erens et al., 2014). A total of 1715 women aged 45–60 in England participated in Natsal-3; 1646 had data available on sexual activity in the past year, of whom 1228 were sexually-active and provided data on sexual function.

As a survey designed to be broadly representative of the general population, and given the low estimated prevalence of HIV in Britain (Brown et al., 2017) and that just three female participants in a random subsample of 2,665 sexually-experienced women aged 16–44 years tested positive for HIV antibody (Sonnenberg et al., 2013), we assume the number of HIV-positive participants in the Natsal-3 sample used for this study to be negligible.

#### The PRIME study

The PRIME study is an observational study exploring the impact of the menopause on the health and wellbeing of women living with HIV (Tariq, Burns, Gilson, & Sabin, 2019). The study recruited 869 women living with HIV aged 45–60 attending one of 21 National Health Service (NHS) HIV clinics in England between February 2016 and June 2017. Participants completed self-administered paper-based questionnaires (including validated questions on menopausal status and symptomatology, as well as sexual function), supplemented with routine clinical data. This analysis draws upon data from participants who reported being sexually active in the year prior to completing the questionnaire *and* reported data on sexual function, (*n* = 386/709, 54.4%).

### Eligibility

The analysis was restricted to women aged 45–60 to capture those undergoing menopausal transition and to minimise the risk of multicollinearity between age and menopause status (Taylor et al., 2015). The measure of sexual function used in this analysis is only validated in sexually-active women (Mitchell, Ploubidis, Datta, & Wellings, 2012), hence we restricted our analyses to those reporting sexual activity (defined as vaginal, oral, or anal sexual intercourse) in the year prior to questionnaire completion. Women from both datasets were excluded from analyses if they had missing sexual function data as this occurred infrequently (<5% of each sample).

### Variables

Where there were differences in the wording and/or response options of some variables, then consensus was reached among authors (CM, NT, ST) on the best way to regroup/recode (see supplementary table). There were no differences in wording of our outcome variable (Natsal SF) and key potential confounding variables such as ethnicity and depression.

#### Natsal-SF

Sexual function was assessed with the Natsal Sexual Function (Natsal-SF) questionnaire, a brief 17-item measure of sexual function in the last year, addressing specific sexual problems, sexual partnership-specific issues, a self-appraisal of participant’s sex life, and whether they had sought help/advice about their sex life. Using this psychometrically-validated and reliable measure of sexual function enables this study to conceptualise sexual function as the extent to which an individual is able to participate in and enjoy a sexual relationship rather than focusing simply on physiological aspects of sexual response and/or clinical diagnoses of dysfunction. However, Natsal-SF measure scores are associated with clinical status (defined as receipt of treatment for sexual difficulties) (Mitchell et al., 2012). The Natsal-SF was used in both Natsal-3 and the PRIME Study, with wording unchanged between both questionnaires. Item non-response in both Natsal-3 and PRIME was generally low (<5% in Natsal-3; PRIME ∼7%).

The Natsal-SF allows an overall sexual function score to be derived for sexually-active respondents, which we use as our primary outcome, alongside the eight individual sexual problems asked about. We used a previously published scoring method (Jones et al., 2015), in which scores range between 0 and 38, with *higher scores* indicating *lower* sexual function. This score was then dichotomised using a score ≥ 13.44 (corresponding to the upper quintile (80th percentile) in the Natsal-3 sample) to denote relatively poor sexual function (Mitchell et al., 2013).

#### Menopausal status

Menopausal status was categorised as a binary variable, i.e., “premenopausal” and “postmenopausal” from data on self-reported menstrual pattern in each survey. In Natsal-3, women were categorised as “menopausal” if they were aged ≥45 years and had been amenorrheic for over one year (Mitchell et al., 2013), while in the PRIME Study, women reporting ≥12 months of amenorrhea were categorised as “postmenopausal”. All other women in both surveys were categorised as “not menopausal” in this study. Women were excluded from participating in the PRIME study if they were on hormonal contraception, were pregnant or breastfeeding, had a history of hysterectomy or bilateral oophorectomy, or if their last menstrual period was >60 months prior. This was not the case in Natsal-3 as these data were not available.

#### CD4 count and HIV viral load

Nadir and most recent CD4 count, and HIV viral load were extracted from routine clinical data where possible, and available for 62.4%, 89.0% and 95.6% of PRIME participants respectively.

### Statistical analysis

Analyses were mainly conducted in SPSS 24 (IBM Corp, released 2016, IBM SPSS Statistics for Mac, Version 24.0, Armonk, NY), using complex survey methods to take account of Natsal-3’s design (including that data were weighted to be broadly representative of the general population) and that recruitment of PRIME participants was clustered by clinic. One exception was that we used Stata version 15.1 (StataCorp LLC, College Station, TX) for the propensity score matching.

We present percentages with associated 95% confidence intervals (CI), as well as medians and interquartile ranges (IQR). Chi-squared tests were used to identify statistically significant associations (*p *< 0.05) between HIV status (i.e., whether in Natsal-3 or PRIME) and sexual function outcome variables. We identified a number of confounding variables *a priori* from the literature including age, ethnicity, number of reported chronic medical conditions, and depression (screened using the Patient Health Questionnaire-2 or PHQ-2 (Arroll et al., 2010; Arroll, Khin, & Kerse, 2003)). We used multivariable logistic regression models to calculate the odds of having (e.g.,) relatively poor sexual function in relation to not, for women with HIV relative to women without HIV adjusting for these confounders.

Given differences in the ethnic composition of Natsal-3 and PRIME samples, we used propensity score adjustment in an effort to further reduce the effects of confounding (Austin, 2011). We used Stata’s “pscore” function to derive a propensity score based on all variables that potentially confounded the relationship between sexual function score and HIV status, listed above, so including but not limited to ethnicity. We then fitted a multivariable logistic regression model, as described above but additionally included the propensity score as a covariate to see if this attenuated the association between HIV status and sexual function.

Finally, we included HIV/menopause status as an interaction term in the multivariable model of overall sexual function to explore whether, and if so, how, this association differed by HIV status.

### Ethics committee approval

The PRIME Study had ethical approval from the South East Coast-Surrey Research Ethics Committee (REF 15/0735). The Natsal-3 study was approved by the Oxfordshire Research Ethics Committee A (reference: 09/H0604/27).

## Results

### Sample characteristics

Women living with HIV were slightly younger than Natsal-3 participants (median age: 49 vs. 51, Table 1). Approximately 70% of women living with HIV were black African, whereas 87.5% of Natsal-3 participants were white British. The majority of women living with HIV and Natsal-3 participants were in an ongoing relationship (85.1% and 88.7%, respectively, *p *= 0.74). Natsal-3 participants were more likely than women living with HIV to be postmenopausal (56.3% vs. 27.8%, *p *< 0.001). Almost all women identified as heterosexual (97.9% and 99.2% in Natsal-3 and PRIME respectively).

**Table 1.** Sample characteristics of sexually-active participants in Natsal-3 and PRIME.

	Natsal-3 (HIV-)	PRIME (HIV+)	*p*-value^d^
	*N* = 1228^a^, 1677^b^	*N* = 386	
	n (%)^c^	n (%)	
*Age (years)*^e^			
Median (IQR)	51 (48–55)	49 (47–52)	<0.001
*Ethnicity*			
White British	1464 (87.5)	35 (9.3)	<0.001
Black African	23 (1.4)	260 (69.1)	
White Other	62 (3.7)	20 (5.3)	
Black Other	33 (2.0)	37 (9.8)	
Other	91 (5.4)	24 (6.4)	
*Education*			
No qualifications	216 (13.0)	33 (8.9)	<0.001
O Levels/GCSEs^f^	647 (38.9)	76 (20.5)	
A Levels^g^	404 (24.3)	78 (21.1)	
University degree or above	396 (23.8)	183 (49.5)	
*Employment status*			
Full/part-time employment	1235 (73.8)	280 (74.7)	0.74
No employment	439 (26.2)	95 (25.3)	
*Ongoing relationship*			
No	189 (11.3)	57 (14.9)	0.73
Yes	1488 (88.7)	326 (85.1)	
**Menopause status**			
Pre-menopausal	727 (43.7)	278 (72.2)	<0.001
Post-menopausal	936 (56.3)	107 (27.8)	
*Number of chronic conditions (other than HIV)*			
None	1165 (70.0)	201 (53.2)	<0.001
1	404 (24.2)	130 (34.4)	
2	77 (4.6)	41 (10.8)	
3	16 (1.0)	5 (1.3)	
≥4	4 (0.2)	1 (0.3)	
*Depressive symptoms (PHQ-2)*			
No (score < 3)	1496 (89.2)	288 (77.6)	<0.001
Yes (score ≥ 3)	181 (10.8)	83 (22.4)	
*Current smoker*			
No	1346 (80.3)	344 (91.7)	<0.001
Yes	331 (19.7)	31 (8.3)	
*Recreational drug use*^h^			
No	1645 (98.1)	366 (96.8)	0.24
Yes	32 (1.9)	12 (3.2)	

PRIME data were not weighted. Where the numerators do not add up to the corresponding denominator, this is due to missing data. IQR, interquartile range; PHQ, Patient Health Questionnaire.

^a^Unweighted denominator.

^b^Weighted denominator.

^c^Weighted numerator.

^d^Chi-squared *p*-value for all associations except age.

^e^Mann-Whitney U median test.

^f^Equivalent to US Grade 10.

^g^Equivalent to US Grade 12.

^h^PRIME asked about recreational drug use in the past three months, whereas Natsal-3 asked in the past year.

Compared to Natsal-3 participants, women living with HIV were more likely to report ≥1 chronic condition other than HIV (46.8% vs. 30.0%, respectively, *p *< 0.001) and score above the cut-off point for depression (22.4% vs. 10.8%, *p *< 0.001). The majority of women living with HIV had an HIV viral load <40 copies/ml (*n* = 235/256, 91.8%); median last CD4 count was 632 cells/mm^3^ (IQR 509-830 cells/mm^3^). The mean time since HIV diagnosis was 13.8 years (standard deviation 6.9 years) with a median nadir CD4 count of 189 cells/mm^3^ (IQR 78-288 cells/mm^3^).

### Overall sexual function

Compared to Natsal-3 participants, women living with HIV had lower sexual function according to the Natsal-SF measure (median score: 8.44 vs. 11.59, *p *< 0.001), with women living with HIV more likely to score above the cut-off for low sexual function (22.9% in Natsal-3 participants vs. 44.6% in women living with HIV, *p *< 0.001, Table 2). This remained the case after adjustment (adjusted odds ratio (AOR) 3.75 [2.15–6.56]). Using a model additionally including the propensity score, and the association between HIV status and sexual function was attenuated but remained statistically significant (AOR 2.43 [1.68–3.51]).

**Table 2.** Prevalence and odds ratios of reporting sexual function problems by sexually-active participants in Natsal-3 and PRIME^a^.

Sexual Problems	Natsal-3 (HIV-) N = 1228^b^, 1677^c^ % (95% CI)	PRIME (HIV+) N = 386 % (95% CI)	*p-*value^d^	Crude OR (95% CI)	AOR^e^ (95% CI)
Lacked interest in sex	38.3% (36.1–40.5)	48.4% (41.4–56.6)	0.007	1.51 (1.12–2.04)	2.30 (1.30–4.07)
Lacked enjoyment in sex	13.1% (11.6–14.8)	31.6% (27.4–36.1)	<0.001	3.06 (2.39–3.91)	3.50 (1.94–6.30)
Felt anxious during sex	3.5% (2.6–4.6)	16.1% (12.6–20.3)	<0.001	5.34 (3.56–8.00)	4.01 (2.24–7.16)
Physical pain as a result of sex	7.5% (6.3–8.9)	15.3% (12.8–18.2)	<0.001	2.23 (1.69–2.95)	2.71 (1.83–4.01)
No excitement/ arousal during sex	8.7% (7.5–10.1)	28.8% (23.2–33.3)	<0.001	4.07 (3.02–5.49)	3.17 (1.84–5.44)
No orgasm/ took a long time to reach orgasm despite arousal	14.9% (13.2–16.6)	31.1% (26.7–35.8)	<0.001	2.59 (2.01–3.32)	2.82 (1.86–4.28)
Reached orgasm too quickly	2.4% (1.8–3.3)	7.4% (4.6–11.2)	<0.001	3.15 (1.79–5.54)	2.20 (0.67–7.26)
Vaginal dryness	17.2% (15.5–19.1)	28.5% (24.4–33.0)	<0.001	1.91 (1.50–2.45)	2.44 (1.47–4.06)
Experienced at least one problem	54.3% (52.1–56.5)	68.7% (63.8–73.1)	<0.001	1.84 (1.46–2.33)	2.44 (1.49–4.00)
Overall sexual function^f^ Low sexual function	22.9% (21.0-25.0)	44.6% (37.3–52.2)	<0.001	2.71 (1.96–3.75)	3.75 (2.15-6.56)

CI, confidence interval; OR, odds ratio; AOR, adjusted odds ratio

^a^Sexually-active participants were those who reported having sex in the past 1 year. Sexual problems were reported from the past year and lasted ≥3 months.

^b^Unweighted denominator.

^c^Weighted denominator.

^d^Chi-squared *p*-value for associations between HIV status and sexual problems variables.

^e^Adjusted for age, ethnicity, ongoing relationship status, depression and number of chronic conditions.

^f^ Score ≥ 13.44 indicated low sexual function.

In univariable analysis among women living with HIV, we found no association between sexual function and nadir CD4 count <350 cells/mm^3^ (OR 0.64 [0.31–1.31]); current CD4 count <500 cells/mm^3^ (OR 0.72 [0.35–1.47]) or having an HIV viral load ≥40 copies/ml (OR 1.02 [0.39–2.70]). Taking women with an HIV diagnosis of <10 years as the reference group, women who had been diagnosed for 10–19 years did not have increased odds of low sexual function (OR 0.96 [0.53–1.72]), however those diagnosed for ≥20 years had over twice the odds of low sexual function (OR 2.44 [1.21–4.90]). This association persisted after adjusting for age (AOR 2.50 [1.22–5.08]).

### Sexual function problems

Both Natsal-3 participants and women living with HIV commonly reported having experienced ≥1 sexual problem lasting ≥3 months in the past year (54.3% and 68.7% respectively, *p *< 0.001, Table 2). In adjusted analyses, women living with HIV were more likely to report ≥1 sexual problem in the past year than Natsal-3 participants (AOR 2.44 [1.49–4.00]).

Each of the eight sexual problems asked about were more commonly reported by women living with HIV. For instance, women living with HIV were more likely than Natsal-3 participants to report a lack of interest in sex (48.4% vs. 38.3%, AOR 2.30 [1.30–4.07]), the most commonly-reported sexual problem in both groups. Vaginal dryness, a common postmenopausal symptom, was nearly twice as common in women living with HIV as Natsal-3 participants (28.5 vs. 17.2%, AOR 2.44 [1.47–4.06]). Women living with HIV were much more likely to report anxiety (16.1% vs. 3.5%, AOR 4.01 [2.24–7.16]), and no excitement or arousal (31.6% vs. 13.1%, AOR 3.50 [1.95–6.30]) during sex.

### Association between menopausal status and sexual function

We found no evidence that low sexual function was associated with menopausal status in the combined Natsal-3 and PRIME sample (AOR 1.18 [0.88–1.59]). When looking at the association between menopausal status and sexual function stratified by HIV status, we found no association between menopausal status and low sexual function among Natsal-3 participants (21.3% (95% CI: 18.6%–24.3%) premenopausal vs. 24.3% (95% CI: 21.8%–26.9%) postmenopausal). Conversely, we did find a statistically significant association between menopausal status and sexual function among women living with HIV, with 53.3% (95% CI: 42.3%–63.9%) of post-menopausal women living with HIV having low sexual function compared to 38.2% (95% CI: 28.9%–48.5%) of premenopausal women living with HIV. However, it is important to note that we found no evidence that HIV status modified the association between menopausal status and low sexual function (*p *= 0.15).

### Seeking help for one’s sex life

Among sexually-active women who reported experiencing ≥1 sexual problem over the past year, women living with HIV were more likely to seek help about their sex life (31.7% vs. 17.1%, *p *< 0.001, Table 3). Women living with HIV were more likely than Natsal-3 participants to seek advice from a family member or friend (12.1% vs. 4.6%, *p *< 0.001), as well as from a sexual health/HIV clinic (12.1% vs. 1.3%, *p *< 0.001). On the other hand, Natsal-3 participants more commonly sought advice from their GP (6.0% vs. 10.4%, *p *= 0.03).

**Table 3.** Advice/care-seeking about one’s sex life among sexually-active participants reporting ≥1 sexual problem(s).

Source of help/advice	Natsal-3 (HIV-) *N* = 656^a^, 910^b^ % (95% CI)	PRIME (HIV+) *N* = 265 % (95% CI)	*p*-value^c^
Family member/friend	4.6% (3.5–6.0)	12.1% (9.5–15.3)	<0.001
Sexual health/ GUM/ STI clinic	1.3% (0.8–2.1)	12.1% (8.7–16.6)	<0.001
Internet	1.8% (1.3–2.6)	7.9% (5.5–11.3)	<0.001
GP/Family doctor	10.4% (8.8–12.3)	6.0% (3.7–9.8)	0.03
Self-help books/information leaflets	1.7% (1.2–2.5)	4.9% (3.2–7.4)	<0.001
Psychiatrist/psychologist	0.5% (0.3–1.0)	1.9% (1.0–3.6)	<0.05
Relationship counsellor	0.7% (0.4–1.3)	1.5% (0.6–3.9)	0.19
Self-help groups	N/A	1.1% (0.3–4.8)	0.02
Other type of clinic/doctor	1.5% (0.8–2.6)	0.8% (0.2–2.9)	0.36
Helpline	N/A	0.4% (0.1–2.7)	0.07
Sought help from at least one source	17.1% (14.9–19.5)	31.7% (26.5–37.4)	<0.001

CI, interquartile range; GUM, genitourinary medicine; STI, sexually transmitted infection; GP, general practitioner.

^a^Unweighted denominator.

^b^Weighted denominator.

^c^Chi-squared test.

## Discussion

Our analysis shows that sexual function problems among sexually-active women aged 45–60 are more common among women living with HIV, compared to a presumed HIV-negative group of women of similar age. Women living with HIV were more likely to exhibit overall low sexual function and to report ≥1 sexual problem lasting ≥3months in the past year. Lack of interest in sex was the most prevalent issue reported by both cohorts of women, although the greatest disparity between the two groups was seen in anxiety and excitement, and arousal. We found that postmenopausal status was associated with low sexual function among women living with HIV, but not among Natsal-3 participants. This contrasts with a previous Natsal-3 analysis which found an association between menopausal status and sexual function among Natsal-3 participants aged 16–74 years, likely a result of the narrower age range used in this present analysis (Mitchell et al., 2013). Compared with Natsal-3 participants, women living with HIV were also more likely to seek informal advice about their sex life from family and friends, and less likely to seek help from healthcare providers outside of sexual health. It is unsurprising that women living with HIV are more likely to seek help within sexual health services as in many parts of the UK, HIV and sexual health services are provided within the same department affording women the opportunity to have sexual health issues addressed.

The effects of living with HIV on women’s sexual well-being are widely recognised (Carter et al., 2017). We found an association between lower sexual function and having been diagnosed with HIV for ≥20 years. Previous research conducted in 1997 has suggested a period of “psychosexual adjustment” after HIV diagnosis (Hankins, Gendron, Tran, Lamping, & Lapointe, 1997). Our data, from the contemporary antiretroviral therapy era, suggest that the impact of HIV diagnosis on sexual function endures longer than previously described. We found no association between sexual function and nadir CD4 count, current CD4 count or HIV viral load, in keeping with other studies (Carter et al., 2017). It is therefore likely that multidimensional contextual factors play an important role in sexual well-being including the emotional impact of HIV diagnosis, experiences of trauma and violence, age, HIV-related stigma, and concerns about HIV transmission (Carter et al., 2017), many of which we did not measure in the PRIME Study. Of particular interest is our finding that the greatest disparities between Natsal-3 and PRIME participants were in reported problems around anxiety and excitement, and arousal. Previous research has identified fear of HIV transmission as an important contributing factor in sexual difficulties (El Fane et al., 2011), and it is reasonable to assume that this impacts both excitement and arousal.

A recent review concludes that the majority of existing work on sex in women living with HIV focuses on either HIV transmission risk or physiological dysfunction, neglecting broader aspects of sexual well-being (Carter et al., 2017). Our study addresses a significant evidence gap on the sexual well-being of midlife women living with HIV and is one of very few comparing sexual function in women with and without HIV. Our findings are consistent with the limited previous literature showing that women living with HIV are more likely to experience diminished sexual function (Denis & Sung-Mook, 2003; Wilson et al., 2010), but builds upon this by focusing on midlife women, a group whose sexual well-being may be compromised by the menopause. Furthermore, by focusing on an older group of women who have been living with HIV for a median of 13.8 years, we highlight the enduring impact of HIV on sexual wellbeing beyond the time of diagnosis.

There were demographic differences between the Natsal-3 and PRIME Study participants; most notably in terms of ethnicity, reported symptoms of depression, and other chronic conditions. We took account of these by using multivariable analyses and propensity score adjustment, however, there may be some residual confounding. Literature on ethnic and cultural differences in women’s sexual function remains limited, with inconsistent findings (Laumann, Paik, & Rosen, 1999; Nazareth, Boynton, & King, 2003; Nicolosi et al., 2004). Furthermore, we did not have data in both datasets on potentially important confounders such as poverty, gender-based violence, and stigma, which could lead to an overestimation of the effect of HIV status on sexual function. Another limitation is that eligibility criteria differed between PRIME and Natsal-3; women were ineligible to participate in PRIME if they reported anything that might disrupt bleeding patterns, including use of hormonal contraception which may have disproportionately excluded women living with HIV who were sexually active. This may have led to an underestimation of low sexual function in this group. Natsal-3 did not share these eligibility criteria, therefore some women may have been misclassified as postmenopausal. We categorised menopausal status on the basis of self-reported menstrual pattern (without biological confirmation). Although this approach is well-validated (Brambilla, Mckinlay, & Johannes, 1994), some women may have been misclassified; it is hard to predict whether and how this might have biased our results. We did not have a detailed menstrual history from Natsal-3 respondents and were unable to differentiate between pre and perimenopausal women. This may have attenuated differences observed between pre and postmenopausal women. We do not know the HIV status of Natsal-3 participants in this analysis. Natsal-3 collected urine samples from a subsample of participants aged 16–44 to test for a range of sexually transmitted infections, including HIV, and of these 2665 women, only three tested positive for HIV antibody, giving an estimated population prevalence of 0·1% (Sonnenberg et al., 2013). Consequently, we believe it is reasonable to assume that Natsal-3 female participants aged 45–60 will have a similarly low HIV prevalence. A further possible limitation is that only sexually active women were eligible to complete the Natsal-SF measure. This is because the majority of items asked about problems that could only arise in the event of sexual activity. Our analysis therefore does not capture prior problems that may have led to cessation of sexual activity, or prevented an individual from becoming sexually active. This may have led to an underestimation of sexual problems, although we are mindful of not assuming that sexual inactivity indicates sexual problems. Moreover, we recognise that sexual activity occurs outside of vaginal, oral, and anal intercourse.

Our detailed analysis of sexual function in midlife women living with HIV is one of the largest internationally, and the first to date in the UK. It is further strengthened by the inclusion of not only a presumed HIV-negative comparison group, but one that is broadly representative of the general population. Moreover, by using the validated Natsal-SF score, we have a multidimensional measure of sexual function that attempts to go beyond a medicalised model of sexual pathology (Carter et al., 2017).

Our analysis highlights the potential impact of HIV on midlife women’s sexual function. There is a paucity of data on the sexual function of women living with HIV (relative to men living with HIV), and even less that focus on older women. Future work in women living with HIV could focus on: biological mechanisms underlying reduced sexual function especially during the menopause transition; exploring the association between sexual function and quality of life; generating a qualitative understanding of sexual well-being and pleasure especially around anxiety, excitement and arousal; and developing interventions that support the sexual well-being of women living with HIV. Finally, in light of data supporting condomless sex in the context of an undetectable HIV viral load as pertaining to HIV transmission, data on sexual function in the era of Undetectable = Untransmittable (U = U) (Rodger et al., 2016) are warranted, especially regarding fears of transmission and the consequent impact on desire, excitement and arousal.

Sexual activity is an important factor in the mental health and quality of life in people *across* the life-course (Hinchliff, Tetley, Lee, & Nazroo, 2018; Lee, Vanhoutte, Nazroo, & Pendleton, 2016). Our findings highlight that sexual problems are commonly reported by midlife women, and that women living with HIV may be at particular risk. UK data suggest that HIV physicians do not routinely ask female patients about sexual function (Bell et al., 2006). Furthermore, a recent survey of UK GPs revealed low levels of confidence in managing menopausal symptoms in women living with HIV (Chirwa, Ma, Guallar, & Tariq, 2017). Therefore, sexual problems in menopausal women living with HIV may go unrecognised and unsupported by healthcare professionals. We advocate that assessment of sexual function be incorporated into the routine HIV clinical care provided to women living with HIV of all ages, and welcome forthcoming UK guidelines on the sexual and reproductive healthcare of people living with HIV which recommend routine annual enquiry about sexual function and menopausal status and symptoms (Waters et al., In press). It is only by thinking beyond the paradigm of HIV transmission risk, and recognising the importance of women’s sexual pleasure *across* the life course, that healthcare providers can support the sexual and reproductive rights of older women living with HIV.

## Supplementary Material

Supplemental Material
