# Supplementary material for: Association of HIV status with sexual function in women aged 45–60 in England: results from two national surveys
Source: AIDS Care. 2019 Aug 14;32(3):286–95. doi: 10.1080/09540121.2019.1653436 (PMC7034538; doi:10.1080/09540121.2019.1653436)
Supplement: Supplemental Material [file CAIC_A_1653436_SM7350.doc]

| Supplementary Table: Variables with wording or response differences and how these were recoded/regrouped | | | | |
| --- | --- | --- | --- | --- |
| **Variable** | **Natsal-3** | **PRIME** | **What we did** | **Comments** |
| **Ethnicity** | *To which of the ethnic groups on this card do you consider you belong?*  **A. White**   1. British 2. Irish 3. Any Other White background   **B. Mixed**   1. White and Black Caribbean 2. White and Black African 3. White and Asian 4. Any Other Mixed background   **C. Asian or Asian British**   1. Indian 2. Pakistani 3. Bangladeshi 4. Any Other Asian background   **D. Black or British Black**   1. Caribbean 2. African 3. Any Other Black background   **E. Chinese or other ethnic group**   1. Chinese 2. Any Other | *What is your ethnic group?*  **A. White**   - English/ Welsh/ Scottish/ Northern Irish/ British - Irish - Gypsy or Irish Traveller - Any Other White background   **B. Mixed/ multiple ethnic groups**   - White and Black Caribbean - White and Black African - White and Asian - Any Other mixed/ multiple ethnic background   **C. Asian/ Asian British**   - Indian - Pakistani - Bangladeshi - Chinese - Any Other Asian background   **D. Black or British Black**   - African - Caribbean - Any Other Black/ African/ Caribbean background   **E. Other ethnic group**   - Arab - Hispanic/ Latino - Any other ethnic group | Re-grouped: “White British”, “Black African”, “White other”, “Black other”, “Other”. | Natsal-3 sample was known to be predominantly white British and the majority of PRIME participants were Black African. |
| **Employment** | Highest occupation code 1: full-time education (incl. if on holiday) 2: government training/employment scheme 3: paid employment (incl. self-employment) 4: waiting to take up paid word already obtained  5: unemployed and registered for benefit  6: unemployed not registered but looking for a job 7: unemployed wanting a job but not looking for a job  8: long-term sickness/disability 9: looking after the home/family 10: doing something else 11: retired 12: temporarily sick/disabled 13: early retirement | *What is your current work situation?*   - Employed or self-employed FULL TIME (at least 30 hours per week) - Employed or self-employed PART TIME (less than 30 hours per week) - Full-time student/ education/ training - Unemployed and registered for benefits - Sick/ disabled (and on benefits) - Looking after home/ family/ dependents full-time - Retired - Other | Regrouped: “Full/part-time employment” and “No employment”. | Specific employment details were not relevant to the analysis. |
| **Education** | *Highest academic qualification*  1: degree level qualification 2: A-levels 3: AS-levels  4: SLC Higher Grade 5: O-level, 1975 or earlier 6: O-level, after 1975 A-C 7: O-level, after 1975 D-E 8: GCSE grades A-C 9: GCSE grades D-G 10: CSE grade 1 11: CSE grade 2-5 12: CSE Ungraded 13: SLC Lower 14: SUPE Lower or Ordinary 15: School certificate 16: Foreign qualification 17: other educational qualification | *What is your highest level of COMPLETED education?*   - Finished education with no qualifications - O levels/ GCSEs (or equivalent qualifications at age 16) - A levels (or equivalent education at age 18) - University degree or above - Other | Regrouped: “No qualifications”, “O Levels/GCSEs”, “A Levels”, “University degree or above”. | The level of education detail in Natsal was not required for this analysis. |

| **Number of chronic conditions** | *Hepatitis B*  Ever diagnosed with hepatitis B  1 = yes  0 = no  *Hypertensi*on 1: yes 0: no  *Diabetes* 1: yes 0: no  *CVD*  Coronary heart disease / angina / other form of heart disease 1: yes 0: no  *Arthritis*  1: yes 0: no  *CVA* Stroke 1: yes 0: no | *Have you ever been diagnosed with any of the following?*   - Hepatitis B - Hepatitis C - High blood pressure - Diabetes - Angina or heart attack - Osteoporosis - Stroke - Breast cancer - None of these | Matched chronic conditions that were asked in both studies (e.g. diabetes) and omitted conditions that were only asked in one (e.g. breast cancer). We included musculoskeletal problems as a condition; in PRIME this was osteoporosis and in Natsal-3 it was arthritis. We created a variable that measured the number of chronic conditions reported (e.g. if a participant reported diabetes and CVD, this was categorised as 2). | Natsal-3 was more comprehensive, and PRIME participants may have experienced additional chronic conditions that were not listed which is a potential limitation. |
| --- | --- | --- | --- | --- |
| **Recreational drug use** | *Taken drugs in last year, including injected drugs*  1: yes  0: no | *In the past 3 MONTHS, have you taken any recreational drugs?* (such as heroin, cocaine, crystal meth, amphetamines [speed] or marijuana, but DOES NOT include any drug taken under a doctor’s instructions)   - Yes - No | Created the variable “Recreational drug use”, coded Yes or No. | The authors acknowledge that this is a limitation but this was a pragmatic approach. |

| **Menopause status** | *Menopausal status* (based on age and last period>1 year indicating menopause)  0: not menopausal  1: menopausal | *Thinking about your periods, when did you last have a period?* Please tick one.   - Less than 1 month ago - 1 to 3 months ago - 4 to 6 months ago - 7 to 9 months ago - 10 to 12 months ago - Between 1 and 2 years ago - More than 2 years ago   *In the past 6 months, have you gone more than two months without having a period?*   - Yes - No - I haven’t had a period in the past 6 months   *In the past 2 YEARS, has your period come early or late by more than a week?*   - Yes - No - I haven’t had a period in the last 2 years - I don’t know/ can’t remember   *If yes, how many times in the past 2 YEARS has this happened?* | In PRIME, pre and peri-menopausal women were combined and categorised as “Pre-menopausal”. | PRIME can distinguish pre-, peri-, and post-menopausal status unlike Natsal which only noted menopausal and not menopausal status.  We made a pragmatic decision and acknowledge this as a limitation. |
| --- | --- | --- | --- | --- |
